# Utilitarian redundancy in local medical systems - theoretical and methodological contributions

**DOI:** 10.1186/s13002-020-00416-x

**Published:** 2020-10-16

**Authors:** Patrícia Muniz de Medeiros, Washington Soares Ferreira Júnior, Fabiane da Silva Queiroz

**Affiliations:** 1 Universidade Federal de Alagoas, Campus de Engenharias e Ciências Agrárias. Br 104, s/n, Rio Largo, Alagoas 57100-000 Brazil; 2 Laboratório de Investigações Bioculturais no Semiárido, Universidade de Pernambuco, BR 203, km 2, S/N, Vila Eduardo, Petrolina, Pernambuco 56328-903 Brazil

**Keywords:** Quantitative Ethnobotany, Data analysis, Local medical systems, Social-ecological systems

## Abstract

The utilitarian redundancy model (URM) is one of the recent contributions to ethnobiology. We argue that URM can be applied to access use-pressure on plant species, the resilience of socioecological systems (e.g., local medical systems), cultural keystone species, and the role of exotic species in social-ecological systems. Based on previous URM studies, we also emphasize the need to differ practical (considering plants and uses that are currently employed) and theoretical (considering both currently employed and potentially employed plants and uses) redundancy. Based on the main applications of the URM, we propose a new index to access redundancy of a therapeutic indication: the Uredit, so that Uredit = NSp + CR, were Uredit is the Utilitarian Redundancy Index for the therapeutic indication; NSp is the total number of species mentioned for the indication, and CR is the species’ contribution to redundancy (in terms of knowledge sharing). The maximum value that the Uredit could reach is twice the number of species employed for the therapeutic indication. We believe that this theoretical and methodological improvement in the model can improve comparisons of redundancy in different social-ecological systems. We also highlight some limitations of the URM (and our Uredit), and we believe that conscious reasons behind people’s decisions should be incorporated into future studies on the subject.

## Background

The utilitarian redundancy model (URM), proposed by Albuquerque and Oliveira [1], was based on the concept of ecological or functional redundancy [2–5] and is one of the recent contributions of ethnobiology both to biocultural conservation and to understand the interactions between people and the environment. Although the model has also been used for other use categories, such as fuelwood [6], most of the investigations on utilitarian redundancy have been applied to local medical systems [7]. These systems involve the body of knowledge and practices related to the perception of diseases and strategies to deal with disease events [8]. Studies on the URM have adopted two different approaches: (1) the search for species that perform the same function in a local medical system (species that treat the same diseases), and (2) the assessment of the degree of redundancy within a therapeutic indication—whether it is treated with few or several species [1, 7].

Two variations of the redundancy model are pointed out in the study of Albuquerque and Oliveira [1]. In the first one, the more species there are for a given function (therapeutic indication), the more distributed is the use-pressure among them, and the more resilient is the medical system. The second variation, however, claims that when a species is preferred, the use-pressure is concentrated on it, even if there are other species in the system for the same function.

Thus, the model of utilitarian redundancy is potentially applied for studies concerning use-pressure on plant resources and resilience of social-ecological systems. Recently, it was suggested that URM could also be applied under an evolutionary point of view, in order to understand the dynamics of local medical systems and what makes some functions more redundant than others [7]. However, despite the potential of URM, the matter has received little attention, being restricted to some investigations that followed the pioneer study mentioned above [9–16]. Díaz-Reviriego [16] proposed an outcome of the URM: the *functional knowledge redundancy*, defined as “the number of species that each distinct group of social actors (population sub-group) knows that treat the same ailment.” Nevertheless, novel outcomes and developments of the model much depend on its methodological development.

Therefore, this paper seeks to theoretically and methodologically contribute to the utilitarian redundancy model. We start by discussing theoretical aspects of the model, and such discussion will be the base for the proposition of a new method for measuring redundancy of therapeutic indications in local medical systems.

## Applications of the URM

Before going further in the theoretical and methodological discussions of the URM, we find it relevant to deepen the model’s main applications. This step is necessary for the reader to understand the bases of our recommendations for the URM. Although we present four applications for the model, two of them will be more deeply developed throughout the text (use pressure and resilience).

### Use pressure

The first significant application of the URM is for studying and inferring about use-pressure. Some ethnobiological studies use the idea of use-pressure without conceptualizing it. We consider use pressure as the demand for a given resource that is effectuated through harvesting. This idea can be based on studies that have investigated the demands of human groups for environmental resources in various aspects [17, 18]. We can imagine use-pressure as a 100 kg iron weight deposited in someone’s back. One average person alone would not stand such a weight. However, with 10 more people, the weight would be distributed, and it would undoubtedly be easy for all of them, together, to hold it. This process is what we (and previous studies) believe happens to plant species that face use-pressure in a scenario in which all species are equally used (see Albuquerque and Oliveira [1]). When a species is significantly more used than the others, we can imagine one person standing the higher portion of the 100 kg iron weight and the others, in the periphery, making little effort to help.

Therefore, redundancy is one of the components to understand plant species’ response to use-pressure, although it cannot be evaluated alone when we seek precise scenarios. To draw a better picture in terms of use-pressure, we would need information such as plant species’ collection frequency, harvesting intensity (amount of resource extracted), used part, and use patterns (e.g., whether bark is removed in longitudinal sections or suffers girdling). Moreover, plant species may respond differently to the same use-pressure, depending on their availability in vegetation areas and the species’ ability (and rate) to recover after damage. Although some studies try to consider some of this information together [19–21], there are several methodological difficulties in establishing a framework for adequately accessing use-pressure or use-pressure consequences to plant populations (e.g., how to effectively measure the amount of resource extracted and plant species’ ability to recover after damage).

### Resilience

The second application of the URM is to contribute to assessing the resilience of social-ecological systems. Resilience has many concepts and interpretations in several scientific fields. We adopt the idea of resilience as the magnitude of disturbance that can be absorbed before the system redefines its structure by changing the variables and processes that control behavior [22]. This concept is commonly used in ecology.

Many ethnobiological studies adopt the ecological concept of resilience to study social-ecological systems (especially local medical systems) [12, 16, 23–26]. However, the transposition of this concept to ethnobiology generated different interpretations in ethnobiological literature. Ferreira Júnior et al. [27] synthesized three interpretations using local medical systems as examples. In the structuralist interpretation, structural changes (e.g., replacement of some medicinal plants by others in a local medical system) may cause the transition of the system to a new regime. The functionalist interpretation considers that if the functions of the local medical system are maintained, this system continues under the same regime (e.g., even the substitution of medicinal plants by allopathy would not be enough to change the system’s domain).

Finally, the processual interpretation offers a “not too wide, not too restrict” view of resilience, claiming that, although structural changes are not sufficient to change the system’s regime, maintaining the system’s functions is not enough for the preservation of such regime. Preservation would be rather linked to the maintenance of the processes that rule the system (e.g., the replacement of a native by an exotic species would not necessarily change the regime, but the substitution of plants by allopathy could change it whenever processes such as knowledge transmission or experimentation were compromised). For this study, we will consider the processual interpretation of resilience.

Redundancy is linked to resilience because, for a given therapeutic indication, whenever a species is lost in the system, others would continue performing the function, and processes related to medicinal plant use would be preserved [27]. However, if a species is unique for a given purpose, its loss could force the system to a rearrangement, introducing, for example, the exclusive use of allopathy and eliminating transmission and experimentation processes related to the therapeutic indication. It means that redundancy may be positively correlated to resilience.

Although redundancy is a critical component, it alone does not explain resiliency. Other aspects, such as knowledge distribution, knowledge transmission, and cultural and symbolic factors need to be evaluated in order to have a better picture of resiliency [27] (e.g., a local medical system may be composed of very redundant targets, but if knowledge transmission paths are blocked, the next generations will not preserve such knowledge).

### Cultural keystone species

The third application of the URM is to help to identify cultural keystone species. The idea of cultural keystone species is derived from the notion of ecological keystone species, i.e., a species that holds the system in check and preferentially consumes species that would otherwise dominate the system [28]. For cultural purposes, a keystone species was defined as “culturally salient species that shape in a major way the cultural identity of a people, as reflected in the fundamental roles these species have in diet, materials, medicine, or spiritual practices” [29].

Therefore, a cultural keystone species is not only a species that is essential in a social-ecological system. Garibaldi and Turner [29] proposed different elements that must be considered when identifying a cultural keystone species. One of those elements is the level of uniqueness in a culture (difficulty to replace the species). One way to evaluate this factor can be by accessing all uses^1^ of a species *x* and observing redundancy for these targets. Uniqueness would be acknowledged if several uses for the species *x* were not redundant.

Besides uniqueness, other factors employed to find cultural keystone species are (1) intensity, type, and multiplicity of use; (2) naming and terminology in a language; (3) role in narratives, ceremonies, or symbolism; (4) persistence and memory of use, and (5) extent to which it provides opportunities for resource acquisition from beyond the territory [29]. Sometimes a species may not be virtually “unique” in a system, but it behaves like it was. In such cases, an interesting aspect of the URM involves the preference of certain items in a redundant use. The model suggests that preferred resources tend to be more harvested than non-preferred ones for the same function [10]. In addition, recent evidence suggests that people tend to choose less preferred resources on redundant uses in the absence of preferred ones, which contributes to the resilience of the system [12]. In this sense, a cultural keystone species could be identified, besides uniqueness, by observing that it is a preferred species in redundant utilitarian categories and that its absence may affect the resilience of the system (people do not select other less preferred redundant species).

### The role of exotic species in local medical systems

Identifying the degree of redundancy within the therapeutic indications, as well as redundant species, may be very relevant to help to understand some aspects of people’s behaviors and cultural evolution. An outstanding example is the entrance of exotic species in local medical systems. Albuquerque [30] suggested that people would use exotic plants to diversify local medical systems by filling blanks that are not occupied (or are poorly occupied) by native species. The author called it the ‘diversification hypothesis’. Subsequent investigations have been favorable to the hypothesis, identifying chemical and utilitarian differences between native and exotic species [31–33].

Redundancy plays a vital role in testing the diversification hypothesis because we would expect to find a higher presence of exotic species in targets with low redundancy in terms of native species (which would be considered as the ‘blanks’ to be filled). Alencar et al. [11] evaluated the role of exotic species in local medical systems through a redundancy perspective, and the authors could not find an association between redundancy and the presence of exotic species. However, the authors did not consider redundancy in terms of native species, but rather the overall redundancy, and we believe that to test the diversification hypothesis, no matter how many exotic species enter to occupy the ‘niche’ (therapeutic indication), it is crucial to evaluate if there are native species’ blanks or not. Therefore, more studies are needed to investigate such a relation.

## Practical redundancy and theoretical redundancy

Ethnobiological literature often distinguishes between known and effectively used resources [34]. One way to refer to those concepts is to separate the practical and theoretical dimensions of local knowledge [35]. Theoretical knowledge is not necessarily being practiced, while practical knowledge emerges when such knowledge is being put into practice. Another way to refer to those concepts is distinguishing stock knowledge (knowledge which is not necessarily being put into practice) and mass knowledge (knowledge that is being put into practice) [30].

While the notion of theoretical knowledge is important under some circumstances, the idea of practical knowledge is more proper to others. For example, studies about use pressure and sustainability should focus on practical rather than theoretical knowledge. If we use our analogy of an iron weight to describe use pressure, it is crucial to evaluate the effect of such weight under people that are carrying it, not considering those that “stand aside only watching.”

For resilience purposes, both theoretical and practical knowledge are important. On the one hand, if a species is lost, a substitute species may leave the theoretical knowledge to enter the practical knowledge. Therefore, all species for a given target must be considered when redundancy is linked to resilience. However, we believe that if a species is part of the practical knowledge for a given therapeutic indication, the chances that it successfully replace a lost species may be higher, since people already count on the species (considering that the species can supply an increasing demand provoked by the loss of one redundant species).

We propose here a distinction between practical redundancy and theoretical redundancy, following the ideas of practical and theoretical knowledge. We believe that, whenever URM is destined to help to evaluate use pressure, practical redundancy should be accessed and, whenever resilience is to be evaluated, both practical and theoretical redundancy should be measured.

Although we consider distinguishing practical and theoretical knowledge, we also believe that such a distinction is far from being easy. Some species may be the first option for treating a given therapeutic indication. Still, if such disease occurs once each 10 years, we may find it challenging to evaluate if it is a case of practical knowledge or not. One way to solve the problem would be to establish a temporal cut-off (i.e., practical knowledge will be considered, for example, only if the species was employed by a person *x* to the therapeutic indication *y* in the last 5 years). But it would be no more than an arbitrary choice.

## The concept of preference and its role in the URM

Preference has been an important concept for URM. Its existence would concentrate use pressure in the preferred species, even if others are known for the same purpose [1]. However, little is discussed about the concept of preference and how (and whether) it fits the idea of the URM.

Ethnobiological literature uses the idea of preference in distinct ways. Most studies use the concept without explaining what is being measured under the ‘preference’ label. Some of them implicitly consider preferred species as a synonym to the most known [36] or the most used [37] species.

However, under our point of view, the most precise concept to preference is “the conscious choice in using a given resource in detriment of another while simultaneously offered” [38]. It means that preference may not be influenced by availability. Preference is conceptually different from the most used species, since someone may prefer a species *x* but not use it due to its low availability in the community. When comparing the most preferred to the most known species, a species *x* may be very popular (known) in a community because of its high availability, although not preferred.

Some studies have found high correlations between the most known and the most preferred species or between the most used and the most preferred species in the context of fuelwood use [39–41]. Nevertheless, high correlations do not mean that these things are the same, and cases in which preference deviates from knowledge and use shall be considered.

The concept of preference proposed by Albuquerque et al. [38] is often employed by other studies [1, 9, 10]. However, we advocate that preference is not the best concept to be associated with URM. If URM is being applied to study use pressure, it is not the preferred species that is going to concentrate such pressure, but the most used species. The preferred species may be so inaccessible (e.g., in an area that is too far from the community) that cost-benefit relations may make people give up harvesting it. A scenario that may be associated with greater use of available but not necessarily preferred resources involves the presence of generalist behaviors in social-ecological systems [42]. This behavior can be favored in situations of environmental scarcity, which leads to the configuration of optimizing what is available, regardless of the quality [42]. The quality and availability of the resources have been important criteria for the local selection of preferred and used plants (see Ferreira Júnior et al., [9], focusing on the preference of medicinal plants; Cavalcanti et al. [41], for preference and use of fuelwood species). Cavalcanti et al. [41] showed that the perceived quality of the fuelwood was the main criterion for the selection of preferred plants, and the availability/accessibility was the main criterion for the use of the resource. In this case, it is possible to think that people can use highly available resources that are not necessarily preferred (high quality) in conditions of environmental scarcity by adopting generalist behaviors. This situation may also emerge in scenarios of use prohibition by external authorities [43], reducing the associations between preference and use. Therefore, the most used species is the one that supports use-pressure, regardless of being preferred or not.

In the context of resilience, studies have employed the idea of preference in medical systems to evaluate people’s strategies to cope with some diseases if the preferred species were not available [9, 12]. Again, we believe that those strategies should be assessed in terms of the most used species, considering that (1) some preferred species may already be unavailable, and (2) if a species is the most used, it means that it plays a significant role in the local medical system, even without being preferred. The more used a species is, the higher the probability that it replaces a redundant species that is being lost. Species may not be used because of its low availability, even if it is a preferred species. In such cases, it may not be a good substitute, since its low availability would not supply the demand for the target.

From now on, when not referring to previous studies, we will employ the term “prioritization” (as a synonym of higher use) instead of preference, to preserve the idea of preference as being different from higher use.

Another important issue is how to measure preference. Some studies on the URM use free listings to access the most preferred species [1]. The most salient species would be the most preferred. Salience is a measure that combines the order in which a species is mentioned in the free listing with the species’ citation frequency. Indeed, studies have found significant correlations between salience and preference [44].

However, it is known that some factors not related to preference may be responsible for the citation of some species in a free listing. Miranda et al. [45], for example, have found that visual stimuli influence people’s responses so that the presence of a species in the place where the interview is being conducted may make people cite it, regardless of its preference. The effect of the context can make a species be considered “preferred” or even “prioritized” only because it was present where most interviews were taking place.

Finally, literature has not developed a quantitative way of measuring redundancy by weighting species according to its utilitarian importance. We will see in the topic 6 that a therapeutic indication for which all species are equally important cannot be considered as redundant as one (with the same number of species) for which one species is highly used and the others are not.

## The role of prioritization and knowledge sharing in the URM

Researches that deal with the URM commonly analyze redundancy based on the percentage of medicinal species known for the therapeutic indications [1, 9, 12, 14, 15]. Therefore, all species have the same weight, regardless of the presence of prioritized species in the medical system. Prioritization (or preference, as used by previous studies on URM) is discussed, but not integrated to the number of redundant species in an index.

When a species (or few species) is prioritized, and many species are poorly cited, redundancy may not be as high as for a therapeutic indication with the same number of species in which there are no preferences (all species are equally popular). Moreover, the low number of citations directed to some species may be due, for example, to their low efficiency, low availability (not supplying the demand) (see Ferreira Júnior and Albuquerque [27]), presence of side effects, or unpleasant taste. In terms of resilience, such factors may decrease chances for a successful replacement. To what concerns conservation, these factors would explain the low use-pressure directed to those species. That is why prioritization and knowledge sharing must be considered when evaluating redundancy.

## Methodological proposition: utilitarian redundancy index for the therapeutic indication (Uredit)

We propose a simple index as a measure of utilitarian redundancy. We are naming it the Utilitarian Redundancy Index for the therapeutic indication (Uredit). We included the term “for the therapeutic indication” because utilitarian redundancy could also be measured for the species, according to the amount of redundancy of the therapeutic indications treated by a given species^2^.

The index is calculated for each therapeutic indication in a system as follows:

$$ \mathrm{Uredit}=\mathrm{NSp}+\mathrm{CR} $$

Were Uredit is the Utilitarian Redundancy Index for the therapeutic indication, NSp is the total number of species mentioned for the indication, and CR is the species’ contribution for generating redundancy (in terms of knowledge sharing).

CR can be calculated as follows:

$$ \frac{\sum Si}{N} $$

Were *Si* represents the number of people who mentioned the species *i* to the treatment of the therapeutic indication *t*, and *N* represents the total number of people interviewed.

To exemplify the calculation of the Uredit, we present the hypothetical case of a local medical system in which 100 people were interviewed. Let us consider a disease that had four plants used to treat it. Plants *a* and *b* were both cited by 50 people for this disease, and plants c and d were cited by 40 and 10 people, respectively. Uredit in this case would be 4 + [(50 + 50 + 40 + 10)/100] = 5.5.

The maximum value that the Uredit could reach is exactly twice the number of species employed for the therapeutic indication. For example, if the target has four species, our Uredit reaches a value of eight when all four species are employed by all 100 respondents (highest sharing and absence of prioritization). In our example above, although the target has four species, Uredit would be 5.5.

The purpose of this calculation is to consider two important aspects mentioned in the previous sections: the number of species employed for a given therapeutic indication and their relative contribution in terms of popularity.

When the therapeutic indication has only one species employed to treat it, there is no redundancy. In such situations, regardless of the degree of sharing (whether the species is known to everyone or only a few respondents), the second component of the index (CR–contribution for redundancy) will not be calculated (no contribution because of no redundancy). It means that Uredit for single-species indications will always be one. This step shall be carefully conducted in data analysis in order to avoid calculating CR for single-species indications.

In most cases, a high correlation may be found between the Uredit and the simple recording of the number of species for each therapeutic indication. It happens because they are not independent since the number of species employed for the therapeutic indication is embedded in the index. However, the use of the number of species as a metric of redundancy could lead to some of the biases discussed throughout this paper (e.g., a therapeutic indication could be considered as redundant even if most species were only known or used by few people).

**Fig. 1:**
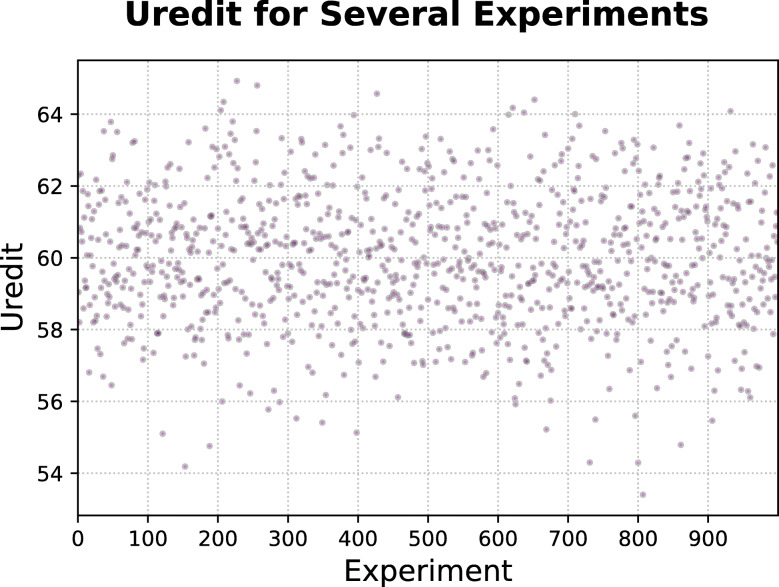
One-thousand simulations of values of the utilitarian redundancy index for the therapeutic indication (Uredit) considering a hypothetical therapeutic indication treated with 40 species and a total of 1000 respondents. Simulated values were the number of people who cited each species for the therapeutic indication

To exemplify the extent to which the Uredit can deviate from the simple recording of the number of species for a therapeutic indication, we ran 1000 simulations of a therapeutic indication’s behavior considering that it has a fixed number of 40 species to treat it and 1000 respondents of a population. The number of respondents that cited each species was randomized in the simulations. Figure 1 shows that Uredit can reach an enormous range of values. Without the index, such variation would converge to a single value: 40 (the total number of species employed for the target.

## Application for the redundancy index: the case of rural communities from Western Bahia

We now add to the simulations performed above a real example of the application of our Uredit. We used databases of three studies conducted in the Western portion of the state of Bahia, Northeastern Brazil [46–48]. The first dataset [46] belongs to an ethnobotanical survey developed in the rural community of Sucruiu, placed in the municipality of Barreiras (25 km from the headquarters of the municipality). The second database [47] belongs to a neighboring community also placed in the municipality of Barreiras (20 km from the headquarters of the municipality), which is called Sucruiuzinho. The third database [48] belongs to the rural community of Morrão de Cima, which is located in the municipality of São Desidério (14 km from the headquarters of the municipality).

All studies were conducted following guidelines developed by the National Health Council by means of the Research Ethics Committee (Resolution 196/96), and the protocol was approved by that committee (CAAE 07488513.4.0000.5026 for Sucruiu and Sucruiuzinho and CAAE 44962515.5.0000.5026 for Morrão de Cima).

They are typical rural communities found in Northeastern Brazil, whose subsistence is based on small-scale agriculture and harvesting of natural resources. Sucruiu has 21 households, and we interviewed 21 family chiefs (men and women). Sucruiuzinho has 20 households, and we interviewed 24 family chiefs. Finally, Morrão de Cima has 30 households, and we interviewed 44 family chiefs. The interviews included questions about medicinal plants they knew and their therapeutic indications (targets). For all plant-therapeutic indication combinations, we asked if the interviewee actually used the species or only knew it.

We considered for the redundancy analysis data for all therapeutic indications cited by at least 10% of the community, to avoid idiosyncratic information. Then we calculated the Uredit for these indications. Calculations of the redundancy index were performed for theoretical redundancy (known and used plants included). Additional information about the communities and study designs can be found in the original papers [46–48].

For the three datasets, CR values were low; most of them reaching less than one. It means that the species’ relative contributions for generating redundancy (in terms of knowledge sharing) are only modest, and the total number of species (from 3 to 75) was responsible for increasing Uredit values.

**Fig. 2:**
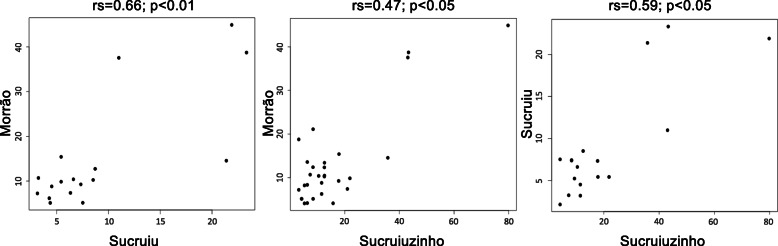
Spearman correlation values for the Uredit of the therapeutic indications treated with medicinal plants in the communities of Sucruiu, Sucruiuzinho, and Morrão de Cima (Western portion of the state of Bahia, Northeastern Brazil)

Considering all the therapeutic indications cited for each community, Uredit means and standard deviations were 11.8 ± 8.4 for Morrão de Cima, 7.0±5.6 for Sucruiu, and 11.5±13 for Sucruiuzinho. A Kruskal-Wallis test found differences between communities to what concerns Uredit values (*H* = 11.36; *p* < 0.01), and the Dunn test found a specific difference between Morrão de Cima and Sucruiu (*p* < 0.01). All other combinations of communities did not present significant differences in terms of Uredit values. Furthermore, we could find significant correlations (Spearman) between the communities in terms of Uredit values (Fig. 2) so that more redundant targets tend to be the same in the three communities (*p* < 0.05 for all correlations). Such correlations were performed only with the therapeutic indications mentioned by both communities in the pairwise evaluation.

**Fig. 3:**
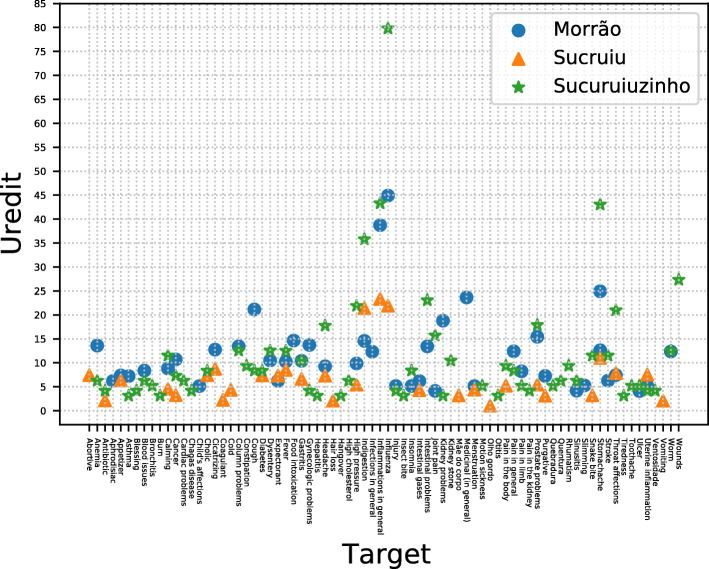
Uredit values for the main therapeutic indications treated with medicinal plants in the communities of Sucruiu, Sucruiuzinho, and Morrão de Cima (Western portion of the state of Bahia, Northeastern Brazil)

General inflammation and influenza were the most redundant targets for all communities. Besides, stomachache also had high Uredit values, with the third-lowest value for Morrão and Sucruiuzinho and the fourth for Sucruiu (Fig. 3).

Considering that, in the three groups evaluated, most therapeutic indications had low to intermediate Uredit values (< 10), and that Uredit values were close to the total number of species (NSp), the indication of these species is poorly shared among the people. A set of existing evidence may suggest that such low redundancy also occurs in other medical systems. The research of Santoro et al. [12] showed that most of the therapeutic indications mentioned by local experts in two human groups showed few redundant species. Additionally, the most redundant indications were those perceived by the specialists with more frequency of occurrence locally. This latter finding was also observed in the study by Nascimento et al. [13], with another human group located in northeastern Brazil. It indicates that the system concentrates a higher amount of plants on a few diseases, which are the most frequent ones.

In addition, Santoro et al. [12] have shown that the sharing of information on plants indicated for the treatment of therapeutic indications is low. In the case, Diaz-Reviriego et al. [16] pointed out that men and women may indicate different plants for the same therapeutic indication in Tsimane’ indigenous groups in the Amazon. Although this difference of indicated plants between groups of people (men and women) would increase the number of redundant species for the therapeutic indications in the system, this also suggests that this knowledge is not shared in these groups.

Based on this evidence, it is possible to suggest that different medical systems (not only in our study) have a low level of redundancy. It would then be interesting to evaluate how these systems with low redundancy respond to disturbances over time. However, a possible response is associated with the presence of a higher number of redundant resources for more frequent therapeutic indications, which could protect the system from disturbances that compromise the treatment of important diseases.

## Criticism, limitations, and perspectives

The utilitarian redundancy model, as well as our redundancy index (Uredit), has some limitations that need to be discussed. First, the only source of information adopted in this kind of approach is the list of plants provided by the interviewees through free-lists, semi-structured interviews, and other types of questionnaires. Therefore, it does not often include people’s perceptions on why certain species are more used than others and whether plant *b* could be a good substitute in case of species *a* loss. We believe that conscious reasons behind people’s decisions should be incorporated into future studies on the subject. A discussion on this issue can be found in a recent paper that introduces the Social-Ecological Theory of Maximization and includes the URM in its theoretical background (see [49]).

We accounted for the role of prioritization and knowledge sharing in our index. Prioritization tends to decrease redundancy (e.g., if only one species is very popular, and the others are rarely cited). Additionally, the low sharing of all the species employed for a therapeutic indication also leads to lower Uredit values. However, our index does not account for differences in use intensity and frequency, as we are only based on popularity. Therefore, if two species are equally popular (cited by the same number of respondents), but one of them is more intensely and frequently consumed, our index is unable to include this type of variation. For this reason, our index has a limited application for conservation studies.

Moreover, we should be cautious when studying pharmacopeias with a high proportion of medicinal plant complexes (mixtures). They can unproperly inflate redundancy. It is possible that a medicinal preparation for a certain therapeutic indication contains more than one species. However, each species may have complementary (and not redundant) functions in the preparation (e.g., providing therapeutic synergism, improving taste, etc.). In such cases, if they enter the calculations as different entities, it could lead to the false premise that one eventually lost species could be replaced by the others.

Leonti et al. [50] commented on the issue of plant complexes as a possible bias to the utilitarian redundancy model. The authors also presented another set of criticism, which, in this case, we believe to be a reductionist view of the model. The authors state that the concept of therapeutic function (here substituted by therapeutic indication) “appears to be conceived as being diagnosis independent classifications into more or less finely tuned etic categories of medical use without contemplating the pharmacology or chemistry of the specific herbal drugs.” They also mention that “for identifying the redundancy of therapeutic functions, the multiple pharmacological mechanisms of action of herbal drugs as well as precise diagnoses, including the identification of pathogenic agents, physiological and histological markers would be necessary.”

However, the reductionist nature of these arguments lies in the fact that in local medical systems, remedies do not need to be pharmacologically interpreted to be used for the same purposes. This perspective has more to do with the researcher’s than with the communities’ point of view. People use medicinal plants based on a whole set of variables (see Caetano et al. [51]) and, among them, their perceived efficiency. Therefore, species with different compounds and mechanisms of action may be perceived as analogous.

## Final remarks

**Fig. 4:**
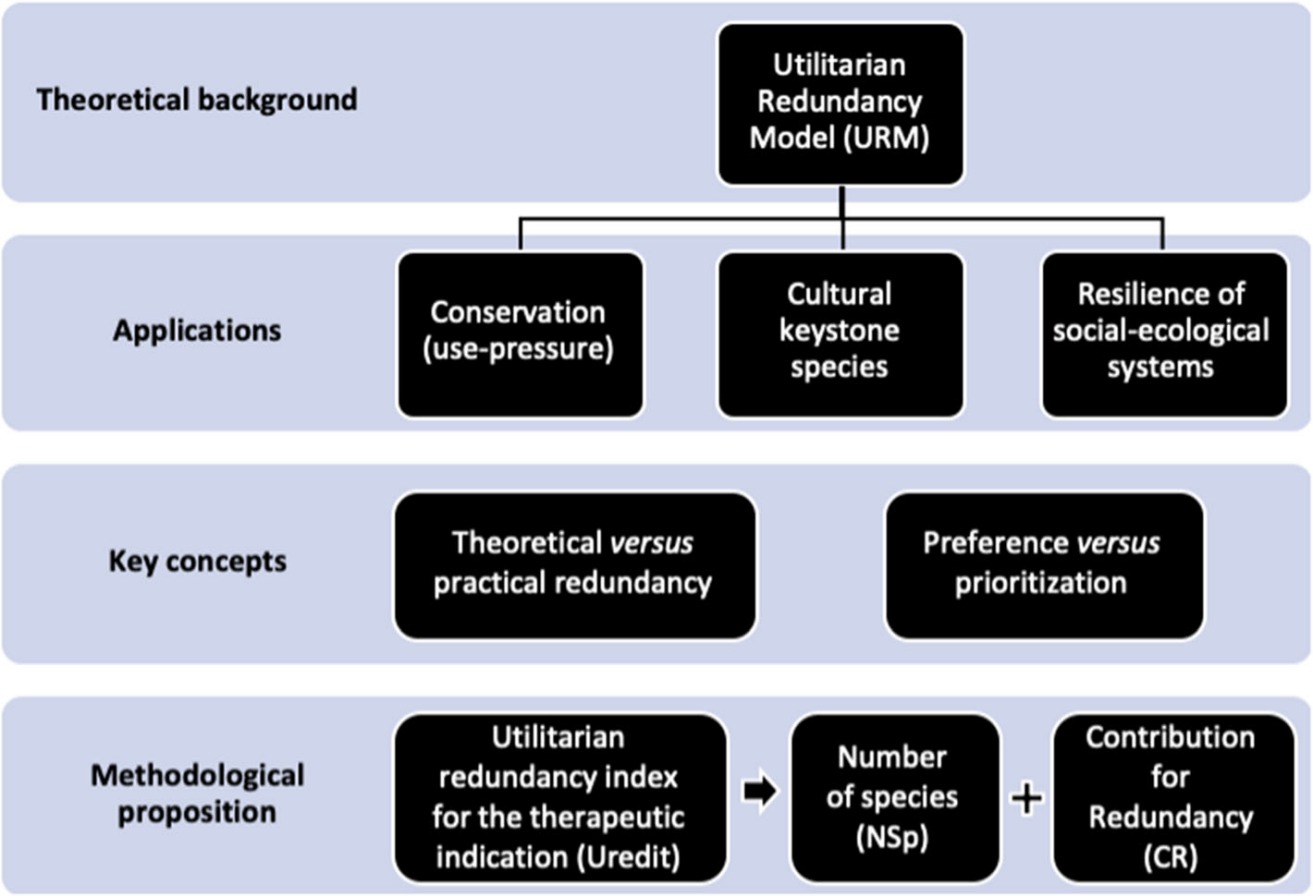
Synthetic scheme of our theoretical and methodological propositions for the utilitarian redundancy model (URM)

In this article, we present some ideas to improve the utilitarian redundancy model, both to indicate reflections on concepts that have been used in the ethnobiological literature and to propose an index to measure redundancy in social-ecological systems based on the presented concepts (see Fig. 4 for a synthesis). We believe that this theoretical and methodological improvement in the model can allow the evaluation and comparison of redundancy in different social-ecological systems so that we can understand (1) what factors affect redundancy in different systems, (2) how system redundancy levels can be modified over time, and (3) how redundancy levels of a system can affect the use pressure of useful species.

## Data Availability

Not applicable—use of secondary data.

## Abbreviations

URM: Utilitarian redundancy model
Uredit: Utilitarian Redundancy Index for the therapeutic indication

## Footnotes

1 For this example, we employed the term “use” instead of “therapeutic indication because the study of cultural keystone species considers all uses performed with a given species in a social-ecological system and not only medicinal uses”.

2 It is not our intention to measure utilitarian redundancy for the species, but rather for the therapeutic indications. Most studies focus on therapeutic indications rather than species, but we believe efforts should be made to develop an index for measuring species’ redundancy.
